# Breast cancer survival and the health system in Brazil: an analysis of public and private healthcare

**DOI:** 10.3389/fonc.2023.927748

**Published:** 2023-05-25

**Authors:** Adriana de Souza Sérgio Ferreira, Jane Rocha Duarte Cintra, Vívian Assis Fayer, Mário Círio Nogueira, Cassimiro Baesso Júnior, Maria Teresa Bustamante-Teixeira, Alfredo Chaoubah, Arthur Duarte Cintra, Caroline Montes Simão, Maximiliano Ribeiro Guerra

**Affiliations:** ^1^ Instituto Oncológico de Juiz de Fora/Hospital 9 de Julho, Departamento de Oncologia Clínica, Juiz de Fora, Brazil; ^2^ Programa de Pós-Graduação em Saúde da Universidade Federal de Juiz de Fora (UFJF), Faculdade de Medicina, Juiz de Fora, Brazil; ^3^ Programa de Pós-Graduação em Saúde Coletiva da Universidade Federal de Juiz de Fora (UFJF), Departamento de Saúde Coletiva, Juiz de Fora, Brazil; ^4^ Hospital Universitário da Universidade Federal de Juiz de Fora (UFJF), Juiz de Fora, Brazil; ^5^ Faculdade de Ciências Médicas e da Saúde de Juiz de Fora (SUPREMA), Juiz de Fora, Brazil

**Keywords:** survival analysis, cohort study, breast neoplasm, prognosis, neoplasm staging

## Abstract

**Background:**

The incidence of breast cancer is increasing globally; however, survival outcomes vary and are lower in developing countries.

**Methods:**

We analyzed the 5- and 10-year survival rates for breast cancer according to the type of healthcare insurance (public *vs*. private) in a referral center for cancer care in the Brazilian southeast region. This hospital-based cohort study included 517 women diagnosed with invasive breast cancer between 2003 and 2005. The Kaplan–Meier method was used to estimate the probability of survival, and the Cox proportional hazards regression model was used to assess prognostic factors.

**Results:**

The 5- and 10-year breast cancer survival rates were as follows: private healthcare service survival rate of 80.6% (95% CI 75.0–85.0) and 71.5% (95% CI 65.4–77.1), respectively, and public healthcare service survival rate of 68.5% (95% CI 62.5–73.8) and 58.5% (95% CI 52.1–64.4), respectively. The main factors associated with the worst prognosis were lymph node involvement in both healthcare services and tumor size >2 cm only in public health services. The use of hormone therapy (private) and radiotherapy (public) was associated with the best survival rates.

**Conclusions:**

The survival discrepancies found between health services can be explained mainly by the difference in the stage of the disease at the time of diagnosis, indicating inequalities in access to the early detection of breast cancer.

## Background

Among the malignant neoplasms that affect women, breast cancer (BC) is associated with high morbidity and mortality worldwide (1), including in Brazil (2). Although BC incidence remains high in high-income countries, these countries have already experienced a tendency to reduce mortality, while middle- and low-income countries show an increased incidence with still high BC mortality (1). Combining population screening with advances in cancer treatment has been identified as an important factor in reducing mortality and the consequent expansion of the number of survivors in high-income countries (3), which reinforces the understanding that the existing differences in availability and access to early cancer diagnosis and treatment contribute to justifying the disparity observed between regions (3, 4).

The relative 5-year BC survival in Brazil increased from 68.7% between 2000 and 2004 to 75.2% between 2010 and 2014, according to surveillance data produced by the CONCORD-3 study. These percentages are lower than those found in North America and Oceania, which have values close to 90% (5). Meanwhile, the 5-year BC survival in Brazil, estimated through hospital-based studies in recent decades, has ranged from 75% to 87% (6–12), while the 10-year BC survival has ranged from 41% to 78.7% (7, 10, 12–14).

Survival analysis is widely used in oncology, especially in BC assessment, because it provides information on the effectiveness of diagnosis and treatment. Furthermore, when performed using population data, it can contribute to identifying specific characteristics of disease behavior and its prognostic factors (14, 15).

Prognostic factors are fundamental in supporting the adoption of adequate criteria for therapeutic approaches. Staging (6, 16, 17), tumor size, lymph node status (6, 9, 11, 13), and hormone receptor (HR) status (8, 11) are classic BC prognostic factors, for which there is sufficient scientific evidence to support their strong association with survival. Individual characteristics, such as age at diagnosis (18), race (13, 17, 19), and socioeconomic profile (20, 21), and those related to health services, such as therapeutic approaches (11), access, and type of health services (public or private) (22–25), have also been identified as prognostic factors that can influence BC survival.

Since 1988, Brazil has had a public health system, the Sistema Único de Saúde (SUS), which has recognized health as a right and works through a universal system delineated by territories and hierarchical networks at integrated care levels (26). In addition, private health services (individual or corporate plans) serve 24.5% of the Brazilian population, composed mainly of formal workers who are part of corporate health insurance plans (26, 27). In the context of BC, some evaluations indicate that there are differences between public and private health services regarding diagnosis and treatment in Brazil, suggesting that public health service users present with a more advanced stage of BC at diagnosis and, consequently, have a worse prognosis (12, 25, 28).

Considering the relevant role of health services in cancer care, this study aimed to evaluate the 5- and 10-year BC survival rates according to the type of healthcare service (public *vs*. private) in a reference center in the Zona da Mata Mineira, Minas Gerais State, Brazil.

## Method

This hospital-based cohort study included women diagnosed with BC between January 2003 and December 2005 who underwent surgical and/or complementary treatment (chemotherapy, radiotherapy, or hormone therapy).

All women were assisted at a regional oncology referral center located in the city of Juiz de Fora, Minas Gerais, Brazil. In 2019, the municipality presented a population estimate of 568,873 inhabitants (29), which is the hub of healthcare in the southeast macro-region of Minas Gerais State, comprising 94 municipalities, and considered as a reference in the diagnosis and treatment of several medical specialties (30). The oncology reference center where the study was conducted provides care for the public health system (SUS) and the private healthcare system, which is accredited by the High Complexity Assistance Unit in Oncology (UNACON) with radiotherapy, chemotherapy, and cancer surgery services (31).

Data from the institution’s Hospital Cancer Registry were used to recruit information from patients. Data collection was carried out in a standardized form through a review of medical records by a team previously trained and advised by specialists in pathology and oncology. The retrieval of information on the follow-up of women to access vital status (defined as a determination of date of death or date last known alive) was obtained from the consultation of hospital records, the National Mortality Information System, National Registries of the Deceased (32), and the Individual Taxpayer’s Registry (33), in addition to a telephone call made by the institution’s Hospital Cancer Registry and contact with the patients’ mastologist. Among the 563 women identified, 45 patients with carcinoma *in situ* and one who died less than 30 days after diagnosis were excluded. We analyzed 517 women with invasive cancer, which corresponded to the study population.

The evaluated variables included three dimensions: 1) sociodemographic variables, such as age at diagnosis (<50, 50–60, ≥60 years), skin color (white and non-white), and education level (high/medium, low); 2) tumor aspects: tumor size (≤2 cm, >2 cm), lymph node involvement (present or absent), stage (initial—I, intermediate—II, advanced—III and IV), hormone receptor (positive, negative, not evaluated), and expression of biomarkers such as hormone receptors (HRs) (estrogen and/or progesterone) and human epidermal growth factor receptor-type 2 (HER2) [yes = HR^±^ and Her2^+^ or HR^+^ and Her2^−^; no = HR^−^ and Her2^−^ (triple-negative tumor subtype)]; and 3) characteristics related to health services, such as performing tumor immunohistochemical expression according to the St. Gallen surrogate classification for breast cancer subtypes (34) (done, not done), the average time between diagnosis and first treatment (in days), type of surgery (conservative or radical), and chemotherapy/radiotherapy/hormone therapy use (no, yes). The 5- and 10-year overall BC survival rates were calculated using the time interval between the date of the histopathological report and the date of death or the end of follow-up. Women who remained alive at the end of the follow-up and follow-up losses on the date of the last contact were censored at 60 months (for a 5-year analysis) or 120 months (for a 10-year analysis). All deaths were treated as failures. To assess differences in the distribution of variables, the *χ*² test was used; when necessary, Fisher’s exact test was used. For the survival estimates and their comparison in relation to the studied variables, the Kaplan–Meier method and the log-rank test were used. The Cox proportional hazards regression model was used to assess prognostic factors by computing the hazard ratio (HR) and the corresponding 95% confidence interval (95% CI). The variable selection for the modeling process was based on clinical relevance and its statistical significance in the univariate analysis, considering the same variables for adjustment at 5 and 10 years in both services (public and private). The variables included in the multiple analyses were removed using the backward elimination process.

All analyses were performed using the Stata software package (version 16.0, StataCorp, TX, USA), and the research was approved by the Research Ethics Committee of the Federal University of Juiz de Fora (reference no. 2.038.397). The level of statistical significance was set at 5%. The quality of adjustment was assessed based on the likelihood ratio and overall measure of adjustment quality.

## Results

Of the 517 women evaluated, 248 (48.1%) were assisted in private healthcare and 269 (51.9%) in public healthcare. **Table 1** shows the distribution of women according to the study variables stratified by the type of health service assistance (private or public).

**Table 1:** Distribution of study variables according to the type of healthcare services for the hospital-based cohort.

	Private		Public		
Variables	*n* ^a^	%	*n* ^a^	%	*p* ^b^
	248	48.0	269	52.0	
Status in 10 years					
Alive	182	73.4	164	61.0	<0.001
Dead	66	26.6	105	39.0	
Age					
<50	68	27.4	100	37.2	0.07
50–60	61	24.6	57	21.2	
>60	119	48.0	112	41.6	
Location					
Juiz de Fora	135	54.4	144	53.5	0.88
Other cities	113	45.6	125	46.5	
Skin color					
White	220	88.7	183	68.0	<0.001
Non-white	19	7.7	84	31.2	
Education					
High/medium	164	66.1	92	34.2	<0.001
Low	48	19.4	140	52.0	
Immunohistochemical tumor pattern^c^					
Done	218	87.5	207	76.9	<0.001
Not done	31	12.5	62	23.1	
Tumor size					
≤2 cm	113	45.6	80	29.7	<0.001
>2 cm	126	50.8	179	66.5	
Lymph node involvement					
Negative	145	58.5	132	49.1	0.04
Positive	96	38.7	128	47.6	
Staging					
Initial (I)	83	33.5	46	17.1	<0.001
Intermediate (II)	97	39.1	105	39.0	
Advanced (III and IV)	66	26.6	118	43.9	
Hormone receptor (HR)^d^					
Positive	181	73.0	177	65.8	<0.001
Negative	62	25.0	70	26.0	
Not evaluated	5	2.0	22	8.2	
Expression of biomarkers^e^					
Yes	200	80.7	186	69.1	0.16
No	39	15.7	51	19.0	
Type of surgery					
Conservative	128	51.6	123	45.7	0.19
Radical	112	45.2	136	50.6	
Chemotherapy					
No	96	38.7	91	33.8	0.23
Yes	152	61.3	178	66.2	
Radiotherapy					
No	51	20.6	44	16.4	0.30
Yes	184	74.2	198	73.6	
Hormone therapy					
No	76	30.7	108	40.1	0.03
Yes	172	69.3	161	59.9	

a The total (n; %) of the variables may differ depending on the presence of missing data.

b Chi-square test (or Fisher’s exact test, when indicated); significant if p < 0.05.

c According to St. Gallen surrogate classification for breast cancer subtypes.

d HR: estrogen and/or progesterone hormone receptor.

e Biomarkers: yes = HR^±^ and Her2^+^ or HR^+^ and Her2^−^ (non-triple-negative); no = HR^−^ and Her2^−^ (triple-negative).

The most frequent characteristics found in both services were as follows: age group over 60 years, white skin color, residence in the municipality of the regional oncology referral center (Juiz de Fora), underwent tumor immunohistochemical expression, first treatment within 15 days of diagnosis, intermediate stage (II), tumor size >2 cm, positive hormone receptors, identified biomarker expression, and had undergone chemotherapy, radiotherapy, and hormone therapy (**Table 1**).

Among the women assisted in public healthcare, in the 10-year follow-up, higher percentages of death (39%, *p* < 0.001), low schooling level (63%, *p* < 0.001), non-white skin color (31.5%, *p* < 0.001), tumor size >2 cm (69.1%, *p* < 0.001), lymph node involvement (49.2%, *p* < 0.05), and advanced stage (III and IV) (37.3%, *p* < 0.001) were observed, compared with those assisted by private healthcare.

Considering the characteristics related to healthcare services, having not performed immunohistochemical tumor expression (23.1%, *p* < 0.001) and the use of hormone therapy (40.1%, *p* = 0.04) were more frequent in the public healthcare service than in the private healthcare service. No significant differences were identified in relation to the type of surgery and chemotherapy or radiation therapy according to the type of health service. The average time between diagnosis and first treatment was 11.78 days (95% CI 1.76–21.80) in the private healthcare service and 18.6 days (95% CI 10.34–26.87) in the public healthcare service.

Regarding the biological aspects of the tumor, significant differences were observed between public and private health services concerning positive hormone receptors (73.1% *vs*. 65.8%, *p* = 0.005), whereas no significant difference was observed in the absence of any qualified tumor biomarkers (triple-negative tumor subtype) (21.5% *vs*. 16.3%, *p* = 0.16).

The overall 5-year BC survival rates were 80.6% (95% CI 75.0–85.0) in the private health service and 68.5% in the public health service (95% CI 62.5–73.8), respectively, while the 10-year BC survival rates were 71.5% (95% CI 65.4–77.1) and 58.5% (95% CI 52.1–64.4), respectively.

**Table 2** shows the 5- and 10-year BC survival rates according to the type of healthcare for the study variables. Unadjusted survival function estimates that indicated better survival (*p* < 0.05), at 5 and 10 years, were observed among white women with tumors <2 cm, initial (I) and intermediate (II) stages, positive hormone receptors, and who underwent conservative surgical treatments and hormone therapy. The survival percentages of these characteristics in private health services were greater than those in public health services.

**Table 2:** Distribution of the 5- and 10-year breast cancer survival rates, according to the type of healthcare services and study variables, for the hospital-based cohort.

Variables	%	95% CI	*p* ^a^	%	95% CI	*p* ^a^	%	95% CI	*p* ^a^	%	95% CI	*p* ^a^
	Private						Public					
	5 years			10 years			5 years			10 years		
	80.6	75.0–85.0		71.7	65.4–77.1		68.5	62.5–73.8		58.5	52.1–64.4	
Age												
<50	82.3	70.9–89.5	0.44	77.6	65.5–85.8	0.14	62.9	52.6–71.5	0.09	52.9	42.5–62.3	0.22
50-60	84.9	73.1–91.9		76.8	63.3–85.9		80.3	67.2–88.6		66.2	51.7–77.2	
≥60	77.3	68.5–83.9		65.5	55.7–73.6		67.7	57.7–75.7		59.8	49.5–68.7	
Skin color												
White	82.3	76.6–86.8	0.02	73.4	66.8–78.9	0.02	74.4	67.3–80.2	<0.001	65.2	57.4–71.9	<0.001
Non-white	61.1	35.3–79.2		50	25.9–70.0		55.2	43.8–65.2		43.5	32.5–54.0	
Education												
High/medium	83.3	76.5–88.2	0.66	73.7	66.0–79.9	0.87	72.1	61.5–80.2	0.64	61	49.9–70.4	0.61
Low	80.4	65.8–89.3		72.6	56.6–83.5		68.8	60.1–75.9		57.8	48.8–65.8	
Tumor immunohistochemical expression^b^												
Done	82.6	76.8–87.1	0.02	73	66.3–78.6	0.12	71.9	65.2–77.6	0.01	59.8	52.5–66.3	0.10
Not done	65.9	45.9–80.0		62.5	42.5–77.2		56.6	42.8–68.3		54.4	40.4–66.4	
Tumor size												
≤2 cm	91.0	83.9–95.1	<0.001	83.1	74.5–89.0	<0.001	88.3	78.6–93.7	<0.001	81.1	70.2–88.4	<0.001
>2 cm	73.7	64.9–80.6		63.4	53.9–71.5		61.9	54.3–68.8		50.7	42.7–58.0	
Lymph node involvement												
Negative	87.3	80.7–91.8	<0.001	79.2	71.2–85.2	<0.001	75.2	66.6–81.8	0.10	68.9	59.7–76.3	0.01
Positive	70.9	60.5–79.1		61.4	50.5–70.6		65.5	56.5–73.0		51.3	42.0–59.8	
Staging												
Initial	95.1	87.4–98.1	<0.001	89.5	80.0–94.6	<0.001	90.6	76.9–96.4	<0.001	83.2	67.9–91.6	<0.001
Intermediate	90.6	82.7–94.9		81.1	71.4–87.8		81.2	72.1–87.6		72.9	62.7–80.8	
Advanced	47.7	34.9–59.3		35.8	24.1–47.7		49.2	39.8–57.9		36.7	27.8–45.6	
Hormone receptor (HR)^c^												
Positive	86.5	80.6–90.8	<0.001	78.9	72.0–84.4	<0.001	77.9	71.0–83.5	<0.001	64.1	56.2–70.9	0.03
Negative	65.7	52.3–76.5		53.8	39.7–65.9		57.8	45.3–68.5		56.1	43.5–66.7	
Expression of biomarkers^d^												
Yes	84.7	78.9–89.1	0.02	76.1	69.3–81.6	0.02	75.7	68.8–81.4	0.01	62.5	54.8–69.2	0.13
No	69.9	52.3–82.1		59.9	41.5–74.2		59.9	44.9–71.9		57.6	42.6–69.9	
Type of surgery												
Conservative	88.7	81.7–93.2	0.01	80.7	72.3–86.7	0.01	77.4	68.8–83.9	0.02	66	56.5–73.9	0.05
Radical	77.1	68.1–83.9		66.7	56.7–74.9		64.3	55.5–71.8		55.7	46.6–63.9	
Chemotherapy												
No	81.7	72.2–88.2	0.80	75.4	65.0–83.1	0.42	63.4	52.1–72.7	0.13	53.5	41.9–63.8	0.13
Yes	79.9	72.5–85.5		69.5	61.2–76.4		71.1	63.8–77.2		61	53.2–67.9	
Radiotherapy												
No	86.1	73.0–93.1	0.53	77.2	62.5–86.7	0.59	53.0	36.6–67.1	<0.001	47.3	31.1–61.9	0.01
Yes	82.1	75.7–87.0		73.4	66.0–79.4		76.0	69.4–81.4		64.1	56.8–70.5	
Hormone therapy												
No	59.8	47.5–70.1	<0.001	48.3	36.0–59.6	<0.001	45.7	35.8–55.0	<0.001	40.9	31.1–50.4	<0.001
Yes	89.4	83.7–93.2		81.5	74.5–86.7		83.3	76.5–88.3		70.1	62.1–76.8	

a Log-rank test for each variable.

b According to St. Gallen surrogate classification for breast cancer subtypes.

c HR: estrogen and/or progesterone hormone receptor.

d Biomarkers: yes = HR^±^ and Her2^+^ or HR^+^ and Her2^−^ (non-triple-negative); no = HR^−^ and Her2^−^ (triple-negative).

In the first 5 years of follow-up, the absence of an immunohistochemical profile was associated with lower survival among women from both health service types (**Table 2**). In this condition, the overall 5-year BC survival was 65.9% (95% CI 45.9–80.0, *p* < 0.05) in private health services and 56.6% (95% CI 42.8–68.3, *p* < 0.05) in public health services, in which 23.1% had not performed the immunohistochemical profile, a percentage significantly higher than that found in the private health service (12.5%, **Table 1**).

In the public health service, better 5- and 10-year survival rates were found among women who received radiotherapy (76% and 64%, respectively). Such a difference was not observed in women who were assisted in private health services.

In private health services, the prognostic factors independently associated with the risk of death, at 5 and 10 years, were the stage at diagnosis and the use of hormone therapy. While advanced staging (III and IV) was associated with an increased risk of death (5 years: HR = 11.4; 95% CI 3.75–34.9; 10 years: HR = 7.87; 95% CI 3.40–18.2), the use of hormone therapy had a protective effect (5 years: HR = 0.34; 95% CI 0.17–0.67; 10 years: HR = 0.38; 95% CI 0.21–0.66). In addition, non-white skin color was associated with a higher 10-year risk of death (HR = 1.14; 95% CI 1.01–4.96), with a trend toward a higher 5-year risk (HR = 2.23; 95% CI 0.88–5.67).

Having undergone radical surgery and the absence of hormone receptors and HER2 were associated with a higher risk of death only in the univariate analysis.

Regarding the public health service, an almost five-fold increased risk of death was observed at 5 and 10 years among women with advanced staging (HR = 4.66) compared with the initial staging. A significantly higher risk of death was also found among non-white women, when compared with white women, in 5 years (HR = 1.98; 95% CI 1.19–3.28) and 10 years (HR = 1.91; 95% CI 1.23–2.97). Hormone therapy and radiotherapy maintained the protective effect throughout the entire evaluation period, indicating that women treated with these treatments survived longer than those who were not treated with these therapeutic modalities (hormone therapy—5 years: HR = 0.28; 10 years: HR = 0.44; radiotherapy—5 years: HR = 0.47; 10 years: HR = 0.52). In addition, the use of chemotherapy reduced the risk of death by 50% at 10 years (HR = 0.51; 95% CI 0.29–0.89).

**Figure 1:**
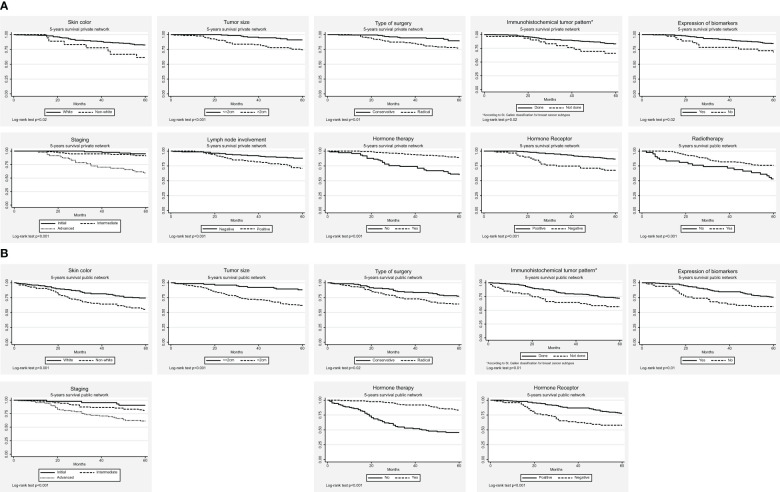
The 5-year breast cancer survival curves, according to the type of healthcare services. **(A)** Private **(B)** Public.

**Figure 2:**
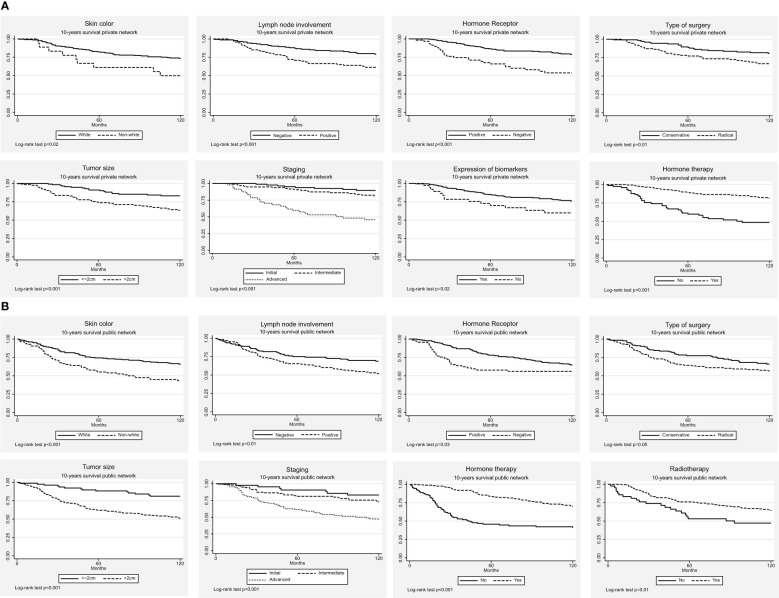
The 10-year breast cancer survival curves, according to the type of healthcare services. **(A)** Private **(B)** Public.

**Table 3** shows the adjusted association measures of the Cox models for the 5- and 10-year BC survival rates according to the type of health services used. The 5- and 10-year overall BC survival curves for significant variables in the univariate analysis (log-rank test) according to the type of health service are illustrated in **Figures 1**, **2**, respectively.

**Table 3:** Adjusted measures of association of the Cox models for 5 and 10-year breast cancer survival rates, according to type of health care service.

	Private					
	5 years			10 years		
Variables	HR^*^	IC 95%	*p*	HR^*^	IC 95%	*p*
Staging						
Initial (I)	1			1		
Intermediate (II)	1.40	0.39–5.04	0.6	1.31	0.52–3.30	0.6
Advanced (III e IV)	11.4	3.75–34.9	<0.001	7.87	3.40–18.2	<0.001
Skin color						
White	1			1		
Non-white	2.23	0.88–5.67	0.09	2.24	1.01–4.96	0.05
Chemotherapy						
No	1			1		
Yes	1.18	0.46–3.02	0.7	1.52	0.70–3.29	0.3
Radiotherapy						
No	1			1		
Yes	0.65	0.24–1.74	0.4	0.75	0.35–1.61	0.5
Hormone therapy						
No	1			1		
Yes	0.34	0.17–0.67	0.002	0.38	0.21–0.66	0.001
	Public					
	5 years			10 years		
Variables	HR^*^	IC 95%	*p*	HR^*^	IC 95%	*p*
Staging						
Initial (I)	1			1		
Intermediate (II)	1.98	0.65–5.97	0.2	1.79	0.75–4.25	0.2
Advanced (III e IV)	4.66	1.63–13.3	0.004	4.66	2.05–10.6	<0.001
Skin color						
White	1			1		
Non-white	1.98	1.19–3.28	0.008	1.91	1.23–2.97	0.004
Chemotherapy						
No	1			1		
Yes	0.62	0.33–1.18	0.2	0.51	0.29–0.89	0.02
Radiotherapy						
No	1			1		
Yes	0.47	0.27–0.82	0.008	0.52	0.31–0.88	0.01
Hormone therapy						
No	1			1		
Yes	0.28	0.16–0.49	<0.001	0.44	0.28–0.71	0.001

HR, hazard ratio. CI, confidence interval.

* Also adjusted for age at diagnosis (continous).

## Discussion

The overall 5- and 10-year BC survival rates were higher in the private healthcare service than in the public healthcare service. The advanced stage at diagnosis was the main factor independently associated with the worst prognosis in both health services. Therapeutic approaches to hormone therapy (in both health services), radiotherapy, and chemotherapy (in the public health service) were associated with better prognosis, whereas non-white race/skin color was associated with worse prognosis in both health services.

According to the international literature, these findings reinforce the differences in access to diagnosis and treatment in more vulnerable populations, such as those found in the Concord study, which was conducted on five continents and showed marked differences in 5-year BC survival between high-income countries (USA and Australia: ~90%) and low-income countries (South Africa: ~40%) (5). Other studies carried out in North American populations also reinforce that advanced stage at diagnosis, low socioeconomic status, and non-white race are associated with lower BC survival and are important determinants for identifying health disparities in this population (35–37).

Brazilian hospital-based studies, which mostly only evaluated women who were assisted in public health services, showed a 5-year BC survival equal to or greater than 75% (6–12), while the 10-year BC survival assessments pointed out a greater range of values, ranging between 41% and 78.7% (7, 10, 12–14). The 5-year BC survival rate found in these studies was higher than that obtained in the present study for women assisted in public health services (68.5%), which was not observed in private health services. To interpret these differences in survival, the higher percentage of characteristics suggestive of better BC prognosis in women who participated in some of these studies, such as earlier stages and positive estrogen and progesterone receptors, must be taken into account (7, 11). In line with the findings of the present study, a study that evaluated health inequities in BC survival in Brazil also observed a worse survival rate in women treated at the public health service compared with those treated at private health services, which was related to advanced staging at BC diagnosis in the public health service (25).

The 10-year overall survival of BC found in the public health service (58.5%) was higher than that observed in a study carried out at the SUS reference center for BC treatment in Joinville, State of Santa Catarina, in the southern region of Brazil (41%) (14). However, it was lower than the 10-year survival found in a university teaching hospital in Belo Horizonte, Minas Gerais (64.5%) (12). Again, these findings may be due to the difference in the distribution of BC stages between regions, since advanced stages were more frequent in Santa Catarina than in Minas Gerais, which concentrated higher percentages of early stages and well-differentiated tumors, characteristics associated with the best survival (12, 14, 23).

Most of the differences found in BC survival according to the type of health service are explained mainly by the difference in the disease stage when women arrived at the health service, indicating inequalities in access to the early detection of BC (12, 23). When the National Cancer Control Policy in Brazil was instituted (38) in 2005, and with its subsequent insertion in the strategic action plan for coping with chronic non-communicable diseases (39), an expansion of access to mammography was observed for the age group of 50 to 69. Another important breakthrough observed was the approval of legal regulations in Brazil, which established a 30-day deadline for diagnostic confirmation and a 60-day deadline to begin treatment (40). In the present study, we observed a high percentage of women who started treatment within 30 days (over 80%) in both types of health services, indicating that access to treatment after BC diagnostic confirmation occurs in a timely manner in both services. However, obstacles in the structure and limited investments in the public cancer care network continue to harm access to early BC diagnosis, recommendations, and timely treatment. These are probably the greatest challenges to enabling a cancer control policy in Brazil that guarantees equity in access to information, tracking, diagnosis, and therapeutic approaches.

The presence of lymph node involvement and tumor size >2 cm are classic prognostic factors associated with a worse prognosis and, consequently, lower survival (6, 7, 9, 13, 14, 18). Lymph node involvement was associated with a higher risk of death in both types of healthcare services, while larger tumor size was associated with a higher risk of death only in the public health service. Women who received treatment in private health service exhibited BC at earlier stages when compared with those treated at the public health service, a finding that corroborates the results of other national studies (12, 23). Such findings suggest greater difficulty in accessing diagnostic confirmation methods and mammographic screening within the Brazilian public health service (41–43), as well as lower percentages of adherence to mammographic screening (43, 44).

The risk of death among non-white women who used the public health service was significantly higher in 5 and 10 years, which can be explained by the higher percentage of advanced stages among women being treated in the public health service. Interestingly, non-white skin color was also associated with the highest risk of death in 10 years among women treated in private health services, showing racial inequalities related to BC control, even in the private network. National and international studies that have investigated BC survival have used skin color as an indirect way of measuring ‘women’s socioeconomic conditions (13, 19, 25, 45–47). Such differences should consider the difficulty in accurately defining skin color due to the intense miscegenation of the Brazilian population and the fact that, in this study, we obtained this information from the individual perceptions of health service professionals. In Brazil, Cabral et al., in their evaluation of Brazilian women with more vulnerable social profiles, such as black skin color and low education, showed long intervals between diagnosis and treatment, regardless of the stage of the disease (48). Our results corroborate those obtained in a study carried out in the southern region of the Mississippi Delta in the United States, which showed high rates of advanced stages in black women in the region, regardless of tumor subtype (20). Racial disparities were also found among African-American women when compared with European-American women, indicating that race/skin color is an important prognostic factor for BC survival. Even when tumor factors are controlled, women of African descent have a higher risk of death from BC, which suggests some secondary effects related to ethnic factors. At diagnosis, these women also have more advanced and aggressive tumors, with a disproportionate chance of survival, most likely due to inadequate access to healthcare as well as socioeconomic disadvantages (35). It is important to highlight that although tumor staging is one of the mediating factors of racial disparities in BC survival identified in several studies, it does not explain all inequalities in prognosis. Other important factors already identified are differences in treatment, the prevalence of comorbidities, and in more recent studies, the interactions between genetic and environmental factors that are mediated by epigenetic modifications (35–37). Another important aspect to emphasize is that racial disparities in BC survival are detected even in models that are also adjusted for socioeconomic variables, indicating that the race/skin color variable is not only a proxy for socioeconomic status, although the latter also plays a role in racial disparities in healthcare (19, 35–37).

The recommendation for hormone therapy was relatively high in both groups (positive hormone receptor status >65% for both health services), which may explain the better survival for women who received this therapeutic modality in both types of health services. However, a higher percentage of unevaluated hormone receptors was found in the public health service than in the private health service (8.2% public *vs*. 2.0% private; *p* = 0.005), which points to disparities in access to diagnostic and therapeutic methods between health services. Hormone therapy was also associated with a better prognosis in a study carried out on Brazilian women by Mendonça et al. (6), De Moraes et al. (7), and Guerra et al. (25).

We verified worse survival for tumors with no expression of any biomarker (triple-negative tumor subtype), corroborating the worst prognosis of this specific tumor subtype and reinforcing the need for a deeper understanding of molecular characteristics to provide more effective treatments. Similar results have been reported by Gonçalves et al. (49) comparing triple-negative and non-triple-negative tumors. In the present study, the distribution of this tumor subtype was similar between both healthcare services, which strengthens the impression that from a biological point of view, the populations under comparison were similar. In the multivariate analysis, we observed that other tumor factors independently influenced survival, such as tumor size in the public health service and lymph node involvement in both health services—factors that are related to the more advanced stage of the disease. These findings were similar to those observed by Fayaz et al. (50) in a 10-year survival study of patients with triple-negative tumors, where staging and lymphovascular invasion were the most relevant prognostic factors for the lowest survival.

Regarding the local therapeutic approach, radiotherapy was associated with better BC survival in 5 and 10 years only at public health services, which is in line with the findings of a study carried out in the western Brazilian Amazon region (11). The distribution of radiotherapy offered in the oncology care network and the displacements needed to arrive at the treatment site can partially explain the difficulties related to accessing this treatment in Brazil (51). Radiotherapy requires complex equipment infrastructure, physical facilities, and highly trained human resources so that it is offered in an appropriate way to the population, conditions that, together, limit its distribution and offer in the SUS network (51). In a study conducted in New Mexico, USA, a two-fold risk of death was identified in women who did not receive radiotherapy compared with those who used the therapies indicated by the National Comprehensive Cancer Network (NCCN) guidelines (52). The use of radiotherapy after conservative surgery reduces the rate of locoregional recurrence and the risk of death from BC, according to a meta-analysis conducted in 2011 by the Early Breast Cancer Trialists’ Collaborative Group (EBCTCG), which included more than 10,000 women with pathologically negative or positive lymph nodes (53). Other studies reinforce the beneficial effect of radiotherapy after mastectomy and adjuvant chemotherapy, even among high-risk cases with lymph node involvement, large tumors, compromised surgical margins, or even in the presence of a combination of risk factors, such as age ≤50 years, triple-negative tumor, high tumor grade, and lymphovascular invasion (54, 55).

For women treated in the public health service, the protective effect of chemotherapy on long-term BC survival (10 years) was also identified. It should be noted that current chemotherapy is more effective, with a reduction in BC mortality with the use of more active regimens, especially in patients with more advanced stages of the disease, when compared with the absence (non-recommendation) of chemotherapy (56).

Although the study has limitations inherent to the use of secondary data, hospital-based cancer registries (HCRs) are recognized as important centers for collecting information on the quality of cancer care. In Brazil, accredited oncology services are required to keep HCRs active and updated, transferring information regarding the care and treatment of cancer patients to the National Cancer Institute (INCA), which uses these data to compare the quality of care provided by oncology services and promote public health policies (57). In the healthcare services where our study was conducted, the HCR has been consolidated and has been in operation since 2000. In addition, a hospital cohort allows greater access to patient follow-up, which contributes to minimizing losses of follow-up that usually occur in cohort studies; a hospital-based cohort allows the adoption of different strategies to retrieve follow-up information, contributing to minimizing the impact of these losses as well as making it possible to recover some selected socioeconomic information through telephone contact, as was carried out for all cases in this study. Although the mean follow-up time was longer in the private network than in the public network, both for the 5-year survival analysis (private: 53.6 months; 95% CI = 51.8–55.3; public: 47.8 months; 95% CI = 45.5–50.0) and 10-year survival analysis (private: 92.4 months; 95% CI = 87.7–97.1; public: 80.5 months; 95% CI = 75.2–85.8), more deaths were identified in the public network than in the private network, and loss to follow-up was not significantly different between health services. As a result of the strategies adopted to retrieve the information, we identified very few losses over 60 months (private: 4.0%; public: 7.4%) and 120 months (private: 18.3%; public: 15.2%).

Furthermore, even in the face of difficulties in evaluating therapeutic recommendations due to the scarcity of available information, the main predictive factors that could influence the use of these therapies were considered during the analysis. On the other hand, the results emphasized the importance of the information produced by health services, which makes it possible to identify the challenges faced, particularly in the public health service responsible for the cancer care of the majority of the Brazilian population (42), as well as makes it possible to produce relevant content to support BC control practices and improve service quality.

## Conclusions

There was a greater BC survival rate in the private healthcare service at 5 and 10 years compared with the public health service, with a worse prognosis related to the advanced stages of the disease and non-white skin color. Hormone treatment contributed to the reduction of the risk of death in both services, pointing to the sustained protective effect in the private network over 10 years, most likely as a result of better guidance of the recommended treatment.

The results of this study strongly emphasize the influence of social inequality on the prognosis of breast cancer in Brazil, highlighting the need, mainly on the part of the public authorities, to reinforce strategies for BC prevention aimed at health education and communication, disease and risk factor surveillance, and early detection, in addition to guaranteeing access to the recommended treatment for all identified cases, especially in the public healthcare network.

## Data availability statement

The raw data supporting the conclusions of this article will be made available by the authors, without undue reservation.

## Ethics statement

This retrospective cohort study was approved by the appropriate ethics committee (Research Ethics Committee of the Universidade Federal de Juiz de Fora, Minas Gerais, Brazil, under reference No. 2.038.397, CAAE: 04575712.4.0000.5147), which allowed dispensing with informed consent for this research, according to national regulations (Res. CNS 196/96). The data were collected from the records of the oncology reference center, and the confidentiality of the participants was protected. We emphasize that all participants are fully anonymous, the database does not contain direct or indirect identifiers, and publication of such data does not compromise anonymity or confidentiality and does not breach local data protection laws.

## Author contributions

AF, VF, JC and MG conceived the study. JC, ADC, VF, CS, CJ and MG were responsible for the data collection, analysis, and interpretation. AF, VF, MN, AC, MB-T and MG designed the analysis and wrote the manuscript. All authors (AF, MG, MB-T, VF, JC, MN, ADC, CS, AC, and CJ) commented on the initial drafts of the manuscript, critically reviewed the manuscript, and read and approved the final version.
